# A Case Report of Abdominal Myoclonus Following Anterior Cerebral Artery Stroke

**DOI:** 10.5334/tohm.1119

**Published:** 2025-12-17

**Authors:** Ksenia Nokhrina, Jana Wold, Meera Raghavan, Guillaume Lamotte

**Affiliations:** 1 Department of Neurology, University of Utah, Salt Lake City, UT, US; 2 George E. Wahlen VA Medical Center, Salt Lake City, UT, US

**Keywords:** stroke, myoclonus, electroencephalogram, post-stroke movement disorders

## Abstract

**Background:**

Post-stroke movement disorders are rare. Abdominal myoclonus following anterior cerebral artery (ACA) infarction has not been previously reported.

**Case report:**

A 75-year-old man presented with acute right-sided weakness and involuntary, rhythmic contractions of the abdominal muscles. Imaging revealed an acute left ACA infarction involving the precentral gyrus. Scalp electroencephalography (EEG) showed no epileptiform discharges. Other secondary causes of myoclonus were ruled out. Abdominal myoclonus resolved after intravenous levetiracetam.

**Discussion:**

This case highlights cortical abdominal myoclonus as a rare manifestation of ACA ischemic stroke. Epileptiform abnormalities on EEG are often not found. Treatment with an antiepileptic medication may resolve the myoclonus.

## Background

Post-stroke movement disorders are a diverse group of hyperkinetic and hypokinetic syndromes, with a reported prevalence of 1.1–3.9% in hospital-based cohorts [1]. These disorders may appear immediately after a stroke or develop after a variable delay, and can be temporary or long-lasting [1,2]. Dystonia, parkinsonism, tremor, and chorea are the most common movement disorders reported after ischemic or hemorrhagic strokes, while myoclonus is a less frequent but clinically important manifestation [1,2].

Myoclonus is defined as a hyperkinetic movement disorder characterized by sudden, brief, involuntary muscle contractions. Although its pathophysiology is not fully understood, proposed mechanisms include cortical hyperexcitability and neurotransmitter imbalances [3]. From a neurophysiological perspective, myoclonus may originate from the cortex, cortico-subcortical circuits (e.g., brainstem), subcortical nonsegmental regions, segmental sites in the brainstem or spinal cord, and the peripheral nervous system [3,4].

Myoclonus is usually classified as either primary or secondary. Primary forms include epileptic, physiologic, essential, and hereditary myoclonus. Secondary, or symptomatic, myoclonus occurs in association with other neurologic or systemic conditions. A wide range of conditions can give rise to secondary myoclonus, including structural brain lesions such as cerebral infarction, inflammatory and infectious diseases (e.g., herpes simplex virus encephalitis), paraneoplastic syndromes, exposure to medications or toxins, metabolic disturbances (e.g., renal failure or hepatic dysfunction), and neurodegenerative disorders such as corticobasal degeneration [3,4].

Lesions involving the cerebellum, pons, midbrain, basal ganglia, and frontoparietal lobes have been reported to cause myoclonus, most often affecting the extremities or the face [5]. In anterior cerebral artery (ACA) infarction, abnormal movements such as asterixis and parkinsonism have been described, with asterixis linked to prefrontal lesions and parkinsonism to involvement of the supplementary motor area [6]. However, post-stroke myoclonus remains rare, and involvement of the abdominal musculature is particularly unusual. We present a case of abdominal myoclonus following a left ACA ischemic stroke.

## Case Report

A 75-year-old right-handed man with a history of coronary artery disease and prior coronary artery bypass grafting presented to the emergency department with acute right-sided weakness and abnormal abdominal movements characterized by involuntary, rhythmic contractions of the abdominal muscles (Video 1). The abnormal movements were confined to the abdominal musculature, predominantly on the right side, and began twelve hours after the onset of weakness. No other body regions were involved. The involuntary movements did not compromise the patient’s respiratory status and were not distractible. The patient was not taking any daily medications. On neurological examination, strength in the right upper and lower extremities was markedly reduced (1/5 on the Medical Research Council scale). General examination demonstrated involuntary, rhythmic, brief, jerky contractions localized to the abdominal muscles, best characterized as myoclonus.

**Video 1:** This video shows a 75-year-old male exhibiting involuntary, rhythmic contractions of the abdominal muscles, predominantly on the right side, consistent with abdominal myoclonus.

The differential diagnosis for the myoclonus included a focal seizure secondary to a structural lesion on the left side of the brain involving the primary motor cortex, as well as other secondary causes of myoclonus, such as drug-induced causes, metabolic disturbances, or, less likely, infectious or neurodegenerative conditions.

**Figure 1:**
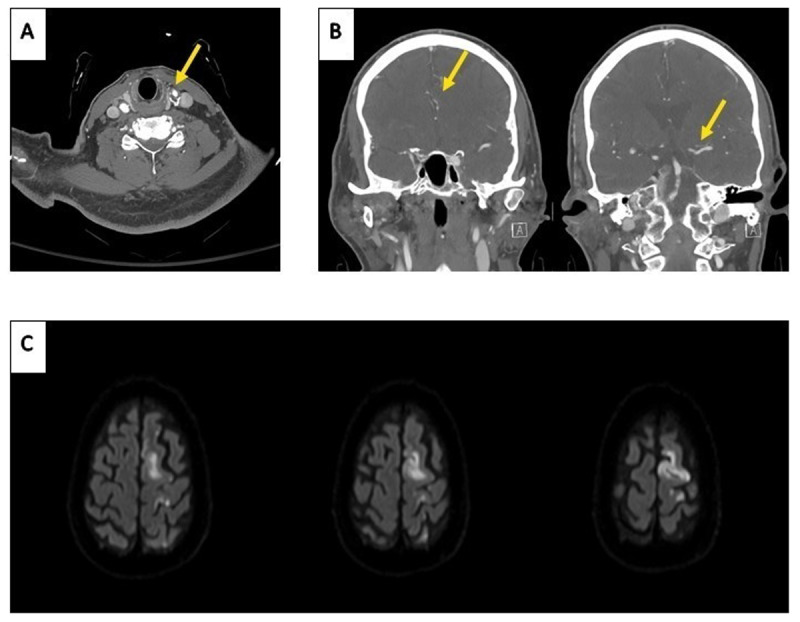
Computed tomography angiography (CTA) of the head and neck and brain magnetic resonance imaging (MRI). **(A)** Axial CTA of the neck showing soft plaque with up to 50% stenosis at the left carotid bulb/proximal left internal carotid artery, **(B)** Coronal CTA of the head showing A2 segment of the left anterior cerebral artery (ACA) stenosis and left medial cerebral artery (MCA) irregularities, **(C)** Diffusion-weighted imaging (DWI) showing acute left anterior cerebral artery (ACA) infarction involving the frontoparietal regions, including the precentral gyrus.

Laboratory tests, including complete blood count, comprehensive metabolic panel, and arterial blood gas analysis, were unremarkable. Computed tomography angiography of the head and neck revealed multifocal atherosclerotic disease with A2 segment of the left anterior cerebral artery (ACA) stenosis and left medial cerebral artery (MCA) irregularities, and 50% stenosis of the left internal carotid artery (ICA) characterized by soft plaque and intraplaque hemorrhage (Figure 1A, 1B). Magnetic resonance imaging (MRI) of the brain without contrast revealed acute infarcts in the left frontoparietal region involving the left precentral gyrus, within the left ACA territory and no evidence of prior strokes (Figure 1C). Specifically, there were no lesions involving the thalamus, brainstem, or frontal lobe. MRI of the cervical and thoracic spine with contrast revealed multilevel spondylosis without spinal cord pathology. The patient was given 2 grams of intravenous levetiracetam, which quickly resolved the abnormal movements. Continuous electroencephalography (EEG) monitoring over 48 hours showed no electrographic seizures or epileptiform discharges. Electrophysiological analysis, including electromyography (EMG) or somatosensory evoke potentials (SSEP), could not be performed because the symptoms resolved rapidly after levetiracetam administration while the patient was in the emergency department.

It was determined that the abdominal myoclonus was due to an acute cortical infarction within the left ACA territory. Because of the symptomatic carotid disease, left ICA stenting was performed during his hospitalization. Levetiracetam was continued for seven days after discharge and then stopped, with no return of myoclonus.

## Discussion

Movement disorders are a relatively uncommon manifestation of acute cerebral infarction. Among them, post-stroke myoclonus remains particularly rare and may be underreported [1,2]. Prior reports have described diaphragmatic myoclonus following left cerebellar hemorrhage, as well as asterixis, often referred to as negative myoclonus, after ACA infarction [6,7]. Myoclonus affecting the abdominal musculature following acute ischemic strokes is exceedingly rare. A case report published in 2015 documented negative axial myoclonus, characterized by decreased cervical and dorsal axial muscle tone upon standing, in patients with thalamic and hippocampal infarcts [8].

The present case is, to our knowledge, the first to describe myoclonus of the abdominal musculature following an ACA territory infarction. Based on the clear history of unilateral motor weakness, acute asymmetric abdominal clonic involuntary movements, and a structural lesion involving the precentral gyrus in the contralateral hemisphere, the etiology of myoclonus in this patient is most consistent with a direct cortical insult. Penfield identified the trunk and abdominal area in the precentral gyrus, medial to the shoulder region and lateral to the hip region in the homunculus [9]. Other causes of secondary myoclonus, including metabolic, infectious, inflammatory and medication induced, were ruled out, and the sudden onset made a neurodegenerative cause unlikely. Diaphragmatic myoclonus was considered; however, due to the rapid resolution of symptoms, we could not perform electrophysiological localization using EMG or video fluoroscopy to differentiate diaphragmatic involvement from abdominal wall myoclonus. Nevertheless, diaphragmatic myoclonus was deemed less likely since there was no evidence of respiratory symptoms. Cases of unilateral abdominal myoclonus have also been reported with lesions in the parietal, occipital, parieto-occipital, and temporo-parietal-occipital lobes [10,11,12]. Importantly, patients with unilateral abdominal myoclonus due to seizures often do not show epileptiform abnormalities on ictal or interictal scalp EEG, likely because of the limited cortical representation of the abdominal wall [13]. The prompt resolution of symptoms after intravenous levetiracetam supports the diagnosis of cortical myoclonus. Pharmacologic treatment of post-stroke myoclonus usually includes clonazepam and sodium valproate, although piracetam and levetiracetam have also shown effectiveness [5].

Limitations include the absence of electrophysiological analysis of the myoclonic jerk, including electromyography and EEG with back-averaging, due to the rapid resolution of symptoms following treatment with levetiracetam. Additionally, we are unable to provide video documentation of the abdominal movements unobstructed by the patient’s gown, which further contributes to the limitations of our study.

## Conclusion

Abdominal myoclonus can be a manifestation of acute ischemic strokes affecting the frontoparietal regions, where the abdominal wall is represented in the motor and somatosensory homunculus. Epileptiform abnormalities on ictal or interictal scalp EEG are often not found. Early treatment with an antiepileptic medication, such as levetiracetam, may lead to the resolution of myoclonus.
